# Accelerated dryland expansion regulates future variability in dryland gross primary production

**DOI:** 10.1038/s41467-020-15515-2

**Published:** 2020-04-03

**Authors:** Jingyu Yao, Heping Liu, Jianping Huang, Zhongming Gao, Guoyin Wang, Dan Li, Haipeng Yu, Xingyuan Chen

**Affiliations:** 1 Department of Civil and Environmental Engineering, Washington State University, Pullman WA, USA; 2 Key Laboratory for Semi-Arid Climate Change of the Ministry of Education, College of Atmospheric Sciences, Lanzhou University, Lanzhou, China; 3 CAS Center for Excellence in Tibetan Plateau Earth Sciences, Beijing, China; 4 Department of Atmospheric and Oceanic Sciences & Institute of Atmospheric Sciences, Fudan University, Shanghai, China; 5 Department of Earth and Environment, Boston University, Boston, MA USA; 6 Northwest Institute of Eco-Environment and Resources, Chinese Academy of Sciences, Lanzhou, China; 7 Atmospheric Sciences and Global Change Division, Pacific Northwest National Laboratory, Richland, WA USA

**Keywords:** Carbon cycle, Climate-change impacts

## Abstract

Drylands cover 41% of Earth’s surface and are the largest source of interannual variability in the global carbon sink. Drylands are projected to experience accelerated expansion over the next century, but the implications of this expansion on variability in gross primary production (GPP) remain elusive. Here we show that by 2100 total dryland GPP will increase by 12 ± 3% relative to the 2000–2014 baseline. Because drylands will largely expand into formerly productive ecosystems, this increase in dryland GPP may not increase global GPP. Further, GPP per unit dryland area will decrease as degradation of historical drylands outpaces the higher GPP of expanded drylands. Dryland expansion and climate-induced conversions among sub-humid, semi-arid, arid, and hyper-arid subtypes will lead to substantial changes in regional and subtype contributions to global dryland GPP variability. Our results highlight the vulnerability of dryland subtypes to more frequent and severe climate extremes and suggest that regional variations will require different mitigation strategies.

## Abstract (web summary)

Earth’s drylands are expected to expand due to climate change, but how this will affect vegetation remains unclear. Here the authors use models to show that despite expansion, primary productivity in drylands is likely to increase through the 21st Century.

## Introduction

Drylands cover ~41% of the Earth’s land surface and support more than 38% of the global population^1^. Grasslands (GRA), shrublands, and savannas (SAVs) are the primary ecosystems of dryland regions^2–4^. Enhanced warming and increasingly frequent and severe droughts^5,6^ threaten the ability of these ecosystems to sequester carbon and maintain biodiversity^7^. Degradation of dryland ecosystems would have strong negative societal and economic impacts, especially in developing countries^8^. A better understanding of the implications of climate-induced changes in dryland ecosystems under future climate scenarios for carbon sink-source dynamics is urgently needed.

Global dryland ecosystems with high biomass turnover rates have accounted for ~40% of the global land net primary production (NPP), dominated the positive global land CO_2_ sink trend, and contributed the largest fractions to the interannual variability (IAV) of net CO_2_ flux over recent decades^9–12^. Such carbon sink variability has been mostly associated with variations in gross primary production (GPP). Using an ensemble of ecosystem and land-surface models and an empirical observation-based product of GPP, Ahlstrom et al.^10^ show that the trend and IAV in CO_2_ sink by terrestrial ecosystems in 1982–2011 were dominated by semiarid ecosystems in response to variations in both precipitation and temperature. As water-stressed biomes, dryland ecosystems are particularly sensitive to drought and wet events, leading to rapid, significant changes in ecosystem structure and functioning and thus large carbon exchange variabilities^13,14^. Such carbon exchange variabilities can even switch between a net sink and a net source from year to year, primarily due to annual precipitation crossing a threshold^15^. Negative dryland GPP anomalies are strongly associated with drought events (warm and dry conditions), whereas positive dryland GPP anomalies are primarily driven by wet events (cool and wet conditions)^16,17^. For example, the 2010–2011 global carbon sink anomaly was mainly ascribed to the enhanced carbon assimilation by Australian dryland ecosystems during wet events^7,11^_._ Such Australian ecosystems have also exhibited repetitive carbon sink episodes^18^, suggesting that dryland ecosystems would exert greater impacts on global carbon cycle variability under future climate change^7,19^.

Model projections show that drylands will experience an accelerated expansion of 11 and 23% by the end of the 21st century under the Representative Concentration Pathway 4.5 (RCP4.5) and RCP8.5 scenarios, respectively, relative to the 1961–1990 baseline^19^. Following the widely used protocol^20,21^_,_ we define drylands as regions with an aridity index (AI) less than 0.65; AI is the ratio of the annual precipitation to potential evapotranspiration (PET). In addition to expansion, degradations and conversions occur across dryland subtypes, which are defined by AI ranges: hyperarid (AI ≤ 0.05), arid (0.05 < AI ≤ 0.2), semiarid (0.2 < AI ≤ 0.5), and dry subhumid (0.5 < AI < 0.65). The expansion usually occurs along the boundaries between the historical drylands (AI < 0.65) and the humid lands (AI ≥ 0.65) by converting humid lands into dryland subtypes as a result of enhanced droughts. Such dryland expansion varies substantially from region to region, with East Asia and North America being particularly prominent. Moreover, climate-induced shifts in ecosystem types also occur across dryland subtypes, and such shifts are highly dynamic even within a given region^19^. However, the implications of such uneven expansion of drylands and dynamic shifts in dryland subtypes for spatiotemporal variability of dryland GPP remain unclear.

Here, we characterize how the projected dryland expansion and degradation lead to shifts in regional and subtype contributions to global dryland GPP variability in response to future climate change under RCP4.5. To quantify regional contributions to the global dryland GPP, we divided the global drylands into eight regions: North America, South America, Europe, Africa, West Asia, East Asia, South Asia, and Australia (Supplementary Table 1 and Supplementary Fig. 1). We used the MODIS-derived GPP data to examine the spatiotemporal variations in the dryland GPP from 2000 to 2014 as the baseline. The MODIS-derived GPP shows good agreement and a linear relation with the FLUXNET-derived GPP at 13 dryland flux sites (“Methods”). These flux sites over drylands are located in North America, Europe, Africa, Asia, and Australia, and their vegetation types are SAV, GRA, croplands (CRO), woody savannas (WSA), and open shrublands (OSH). The good agreements between the annual GPP anomalies from MODIS and those from the flux towers also demonstrate that the MODIS product also captures IAV, consistent with the previous extensive GPP comparisons over global lands^22,23^. Further, we compared spatial and temporal variations in the dryland GPP between the MODIS-derived GPP and the FLUXCOM GPP (“Methods”). Our results confirm the conclusions from previous studies that the MODIS-derived GPP data are reliable in quantifying spatiotemporal variability in global dryland GPP, although underestimations of the MODIS-derived GPP are identified in some regions^24^. Taking into account uncertainties and other factors for the different datasets, MODIS-derived GPP remains one of the most robust datasets to quantify the spatiotemporal variability of GPP at global scales (“Methods”).

## Results

### Global and regional dryland GPP variability (2000–2014)

**Fig. 1:**
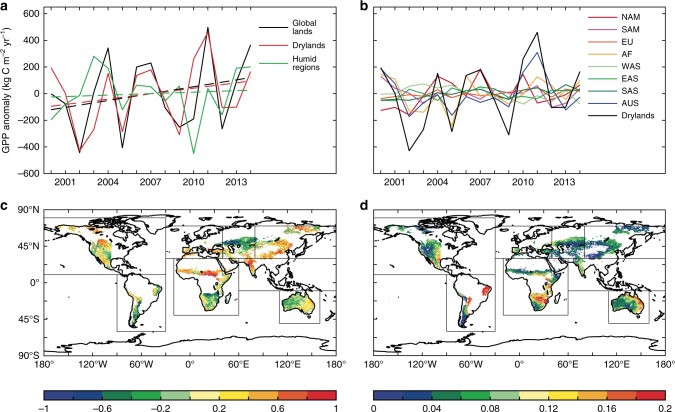
MODIS-derived dryland GPP trend and variations from 2000 to 2014. **a** Variations of the annual gross primary production (GPP) anomaly for the global lands, global drylands, and global humid regions (2000–‍2014). The dotted line denotes the linear trend of GPP. Global drylands played the dominant role in modulating both the trend and the interannual variability (IAV) of the global land GPP, accounting for 79% of the increasing trend and 83% of the IAV of the global land GPP in 2000–2014, although they only contributed to 21% of the global mean GPP. **b** Variations of the annual GPP anomaly over the eight dryland regions in 2000–2014. NAM North America, SAM South America, EU Europe, AF Africa, WAS West Asia, EAS East Asia, SAS South Asia, AUS Australia, Drylands the global drylands. **c** The mean global dryland GPP trend, 2000–2014 (kg C m^−2^ year^−1^). **d** The global dryland GPP IAV, 2000–2014 (kg C m^−2^). The lowest global land GPP anomalies occurred in 2002 and the largest in 2011, which were primarily attributed to the global dryland GPP anomalies in these 2 years (see **a**). The 2011 GPP anomaly was mainly caused by the high GPP in three dryland regions in Australia, Africa, and South America (see **b**), which is consistent with the previous modeling study about the widely reported 2011 record carbon sink. Our analysis indicates that the lowest GPP anomaly in 2002 was associated with the drought event, whereas the largest GPP anomaly in 2011, particularly in Australia and Africa, was associated with the La Niña-induced wet anomaly. The trend of precipitation best explained the trend of the GPP in North America (64%), East Asia (59%), and Africa (68%), whereas the precipitation IAV also best explained the GPP IAV in Australia (79%), South America (72%), and Africa (76%).

**Fig. 2:**
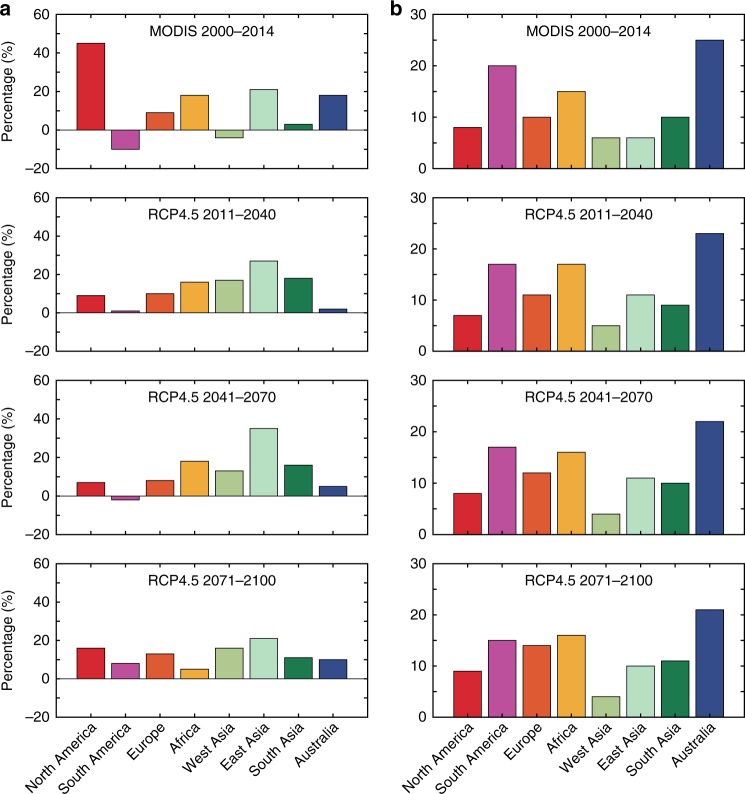
Contributions from the eight regions to global dryland GPP. **a** Gross primary production (GPP) trend and **b** interannual variability (IAV) for 2000–2014 (MODIS) and during the three periods in 2011–2100 (RCP4.5).

Global drylands demonstrated increasing trends and spatiotemporal variations in GPP from 2000 to 2014 (Fig. 1). The largest positive contributions to the increased dryland GPP trend occurred in North America (45%), East Asia (21%), and Africa/Australia (18%) (Figs. 1c and 2; Supplementary Table 2), whereas the largest contributions to the global dryland IAV occurred in Australia (25%), South America (20%), and Africa (15%) (Fig. 1d and Supplementary Table 3). Note that relative changes in trends, IAV, and their contributions are reported with respect to drylands, consistent with the focus of this study; GPP of humid regions (AI >= 0.65) is not discussed hereafter. Among the environmental drivers, precipitation explained 67% of the dryland GPP variations, followed by air temperature, which explained 15% of the dryland GPP variations. The remaining 18% of the dryland GPP variability was explained by a combination of other drivers including soil moisture, vapor pressure deficit (VPD), and PET (“Methods”; Supplementary Figs. 2 and 3). These results are consistent with previous findings that precipitation is the most important driver of dryland vegetation dynamics.

**Fig. 3:**
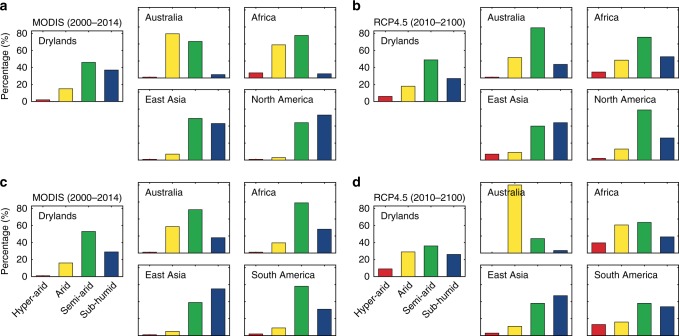
Contributions from the four subtypes to global dryland GPP. **a**, **b** Gross primary production (GPP) trend and **c**, **d** interannual variability (IAV) in 2000–2014 (MODIS) and 2011–2100 (RCP4.5).

Further analysis shows that precipitation and GPP variabilities were significantly correlated in semiarid regions (*R*^2^ = 0.82; *P* < 0.05). Due to their high sensitivity to precipitation (Supplementary Fig. 4), the semiarid regions contributed the most to the trend (46%) and IAV (53%) of the global dryland GPP in 2000–2014 (Fig. 3). The subhumid regions were the second largest contributor to the trend (37%) and IAV (29%) of the global dryland GPP due to the high correlations between GPP and temperature in North America and East Asia with the largest areas of this subtype. When regional and subtype variations are considered jointly, we found that the subhumid (53%) and semiarid (44%) regions in North America, semiarid (49%) and subhumid (43%) regions in East Asia, and semiarid (50%) and arid (39%) regions in Africa primarily contributed to the regional trends (Fig. 3a). The semiarid (51%) and arid (31%) regions in Australia, semiarid (58%) and arid (31%) regions in South America, and semiarid (59%) and subhumid (28%) regions in Africa predominantly regulated the regional IAV during the 2000–2014 period (Fig. 3b).

### Projected dryland GPP in the 21st century under RCP4.5

**Fig. 4:**
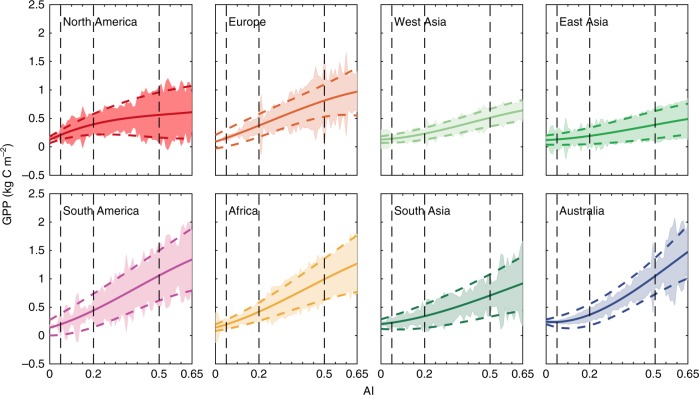
Relationships of GPP to AI in the eight regions (2000–2014). The solid curves denote the fitted relations between gross primary production (GPP) and aridity index (AI) in the eight dryland regions, and the upper and lower dashed curves denote the 95% confidence intervals of the fitted relations. The shaded areas denote one standard deviation. The vertical dashed lines delineate the boundaries between the four dryland subtypes.

**Fig. 5:**
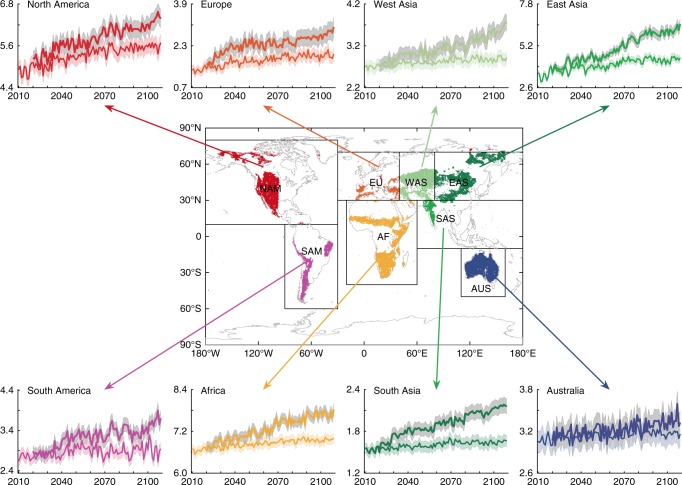
Temporal variations of GPP in the eight regions (2010–2100). The curves with the small and large gross primary production (GPP) in units of Pg C year^−1^ are under RCP4.5 and RCP8.5, respectively. The shaded areas denote 95% confidence intervals.

Warming and drying trends are projected to dominate climate change in the 21st century, accompanied by more frequent extreme climate events^7^. Long-term warming and drying trends would accelerate dryland expansion by converting humid ecosystems into dryland ecosystems and degrading fragile dryland subtypes^19^. To address the questions of whether and how global dryland expansion and dryland subtype conversions would lead to shifts in regional contributions to the global dryland GPP and IAV and changes in subtype contributions across regions, we first adopted the proposition that the relationship between AI and GPP can be used to quantify dryland GPP variability^19^. The tight relationship between GPP and AI is reflected by the high correlation between GPP and AI during 2000–2014 (Supplementary Fig. 5). We examined the relationship between dryland GPP and AI and applied this relationship to quantify the influence of dryland expansion and degradation on dryland GPP. Using the GPP and AI over 2000–2014, we fitted one polynomial function between GPP and AI for each of the eight regions (Fig. 4), with remarkably high correlations (Supplementary Table 4). These high correlations confirm that AI is a good metric to constrain dryland GPP^19^. We also used the two equations of the 95% prediction intervals of the fitted GPP–AI relations (Fig. 4) to calculate the uncertainty in the projected GPP. We then adopted the aforementioned global dryland AI datasets based on the RCP4.5 projections for the period from 2011 to 2100 to drive the fitted functions for each region to estimate variations in the global dryland GPP (Fig. 5). Note that these fitted functions based on the data across the AI range from 0 to 0.65 were only applied to determine the projected GPP over global drylands with the same AI range (i.e., 0 < AI < 0.65). We performed the same calculation under RCP8.5 for comparison. By establishing mapping relations with the 2000–2014 MODIS datasets, we conducted bias corrections on CMIP5 model results^25^. Our 2010–2100 GPP derived from the fitted functions showed promising agreement with the ensemble means of the 15 CMIP5 modeling results in the eight regions during the same period (Supplementary Fig. 6; Supplementary Table 5).

### Influence of dryland expansion and conversions on GPP

**Fig. 6:**
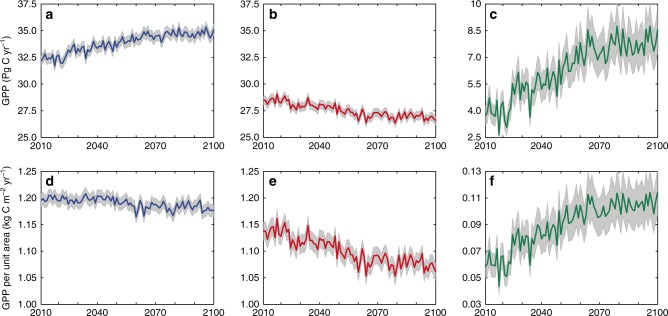
Temporal variations in dryland GPP under RCP4.5 (2010–2100). **a** Temporal variations in the total GPP of the global drylands, including both the historical and expanded drylands. **b** Temporal variations in the total GPP of the historical drylands. **c** Temporal variations in the total GPP of the expanded drylands. **d** Temporal variations in the GPP per unit area in the global drylands, including both historical and expanded drylands. **e** Temporal variations in the GPP per unit area in the historical drylands. **f** Temporal variations in the GPP per unit area in the expanded drylands. The shaded areas denote 95% confidence intervals.

Global dryland GPP will increase by 12 ± 3% by the end of this century under RCP4.5 relative to the 2000–2014 global dryland GPP baseline. The global dryland GPP shows increasing trends and larger IAV values. The global dryland GPP trend and IAV increase by 7 ± 2 and 2 ± 1% in 2011–‍2040, 11 ± 3 and 4 ± 2% in 2041–2070, and 12 ± 3 and 3 ± 2% in 2071–2100, relative to the 2000–2014 global dryland GPP baseline. Such increasing trends and enhanced IAV by the end of this century are attributed to the combined effect of the accelerated dryland expansion, climate-induced shifts of the subtypes within the historical drylands (e.g., primarily drought-induced degradation of drylands), and conversions of drylands to humid lands. East Asia and North America will experience the most significant dryland expansions and changes in subtypes, resulting in large changes in GPP, especially in 2010–2065 (Supplementary Figs. 7 and 8). The drying areas are mainly distributed in Canada, central Africa, western Asia, and northeastern China, with a larger area becoming drier and a smaller area becoming wetter^19^. The widespread expansion-induced GPP increase (0.0661 Pg C year^−1^) substantially surpasses the GPP decrease (−0.0211 Pg C year^−1^) due to the drought-induced degradation of drylands and the wet-induced shrinkage in drylands (Fig. 6), leading to the overall increase in the global dryland GPP by 12 ± 3% at the end of this century (Fig. 5; see more analysis below). Nevertheless, the global dryland GPP per unit area actually decreases at a rate of –0.26 kg C m^−2^ year^−1^ because the decreased GPP in the historical drylands at a rate of −0.83 kg C m^−2^ year^−1^ exceeds the increased GPP in the expanded drylands at a rate of 0.57 kg C m^−2^ year^−1^ (Fig. 6), taking into account the increase in the global dryland areas by 11% at the end of this century under RCP4.5 as compared with the 1961–1990 global dryland baseline^19^. The increasing trends and IAV values will be stabilized after 2065 under RCP4.5 (ref. ^26^).

Substantial shifts in the regional contributions to global dryland GPP trends are observed during different periods of the 21st century. Under the RCP4.5 scenario, the three largest regional contributors to the global dryland GPP trends shift from East Asia (27%), South Asia (18%), and West Asia (17%) in 2011–‍2040 to East Asia (35%), Africa (18%), and South Asia (16%) in 2041–2070, and then to East Asia (21%), West Asia (16%), and North America (16%) in 2071–2100. East Asia emerges as the largest contributor to the trend, exceeding North America and South Asia. Australia, Africa, and South America remain the largest contributors to the IAV, as compared with the 2000–2014 baseline (Fig. 2, Supplementary Fig. 9A). The large GPP IAV in these three regions is likely attributed to the more frequent climate extremes and the high sensitivity of drylands to droughts and precipitation in these regions^12^.

It is observed that there is a shift in the roles of different dryland subtypes in contributing to the global dryland GPP trend and IAV under RCP4.5 (Fig. 3). The largest climate-induced dryland expansion is found to occur in semiarid regions, accounting for almost half of the total dryland expansion^19^, covering more than one-third of the total dryland areas, and contributing almost half of the total global dryland GPP. This explains the increased roles of semiarid ecosystems in regulating the trends (49%) and variability (35%) of the global dryland CO_2_ sink under the future climate change. However, the arid subtype plays an increasing role in regulating the trend (18%) and IAV (29%), whereas the subhumid subtype has a reduced impact on the trend (27%) and IAV (26%) (Fig. 3). The contributions of different subtypes to the trend and IAV also vary across regions. The largest contributions to the semiarid subtype GPP trends occur in East Asia (30%) and Africa (27%), and the largest contributions to the global semiarid subtype GPP IAV occur in Africa (26%) and Australia (24%). The largest contributions to the global arid subtype GPP trends occur in Africa (34%) and Australia (19%), and the largest contributions to the global arid subtype GPP IAV occur in Australia (44%) and Africa (21%), whereas the largest contributions to the global subhumid subtype GPP trends occur in East Asia (31%) and North America (27%) and the largest contributions to the global subhumid subtype GPP IAV occur in East Asia (31%) and South America (16%).

**Fig. 7:**
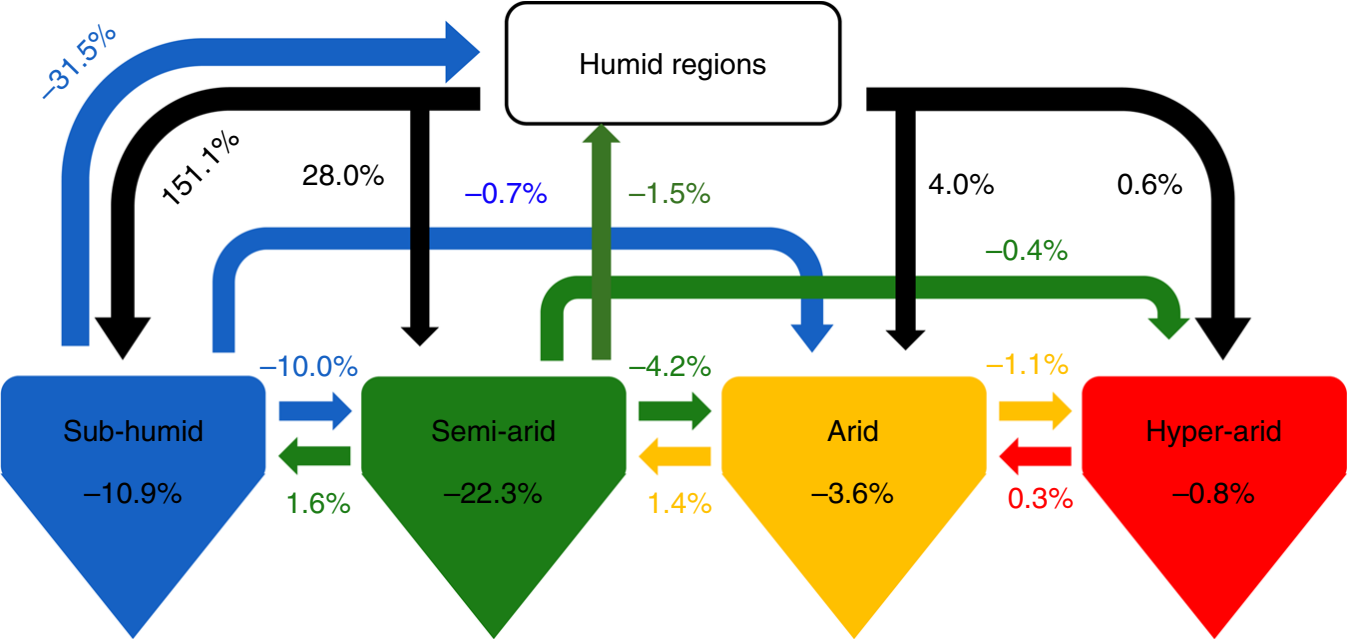
Changes in dryland GPP due to expansion and conversions. It is estimated from our study that the global dryland gross primary production (GPP) will increase by 12% in 2085–‍2100 relative to the 2000–2014 baseline under RCP4.5. Taking 12% as 100 units, the magnitudes in the diagram thus denote the percentage of the contribution caused by each process to this 12% change. The sum of all the numbers in the diagram is equal to 100%. Positive values indicate the increased GPP for the corresponding dryland subtypes and negative values indicate the decreased GPP for the corresponding dryland subtypes. For example, −31.5% means a reduction in the GPP contribution to the 12% change for the subhumid subtype due to its conversion to humid lands, 151.1% means an increase in the GPP contribution to the 12% change for the subhumid subtype due to its conversion of humid lands to the subhumid subtype, and −10.9% means a reduction in the GPP contribution to the 12% change for the subhumid subtype due to its degradation within its subtype. Note that changes in GPP in humid regions are not the focus in this study.

During the 21st century under RCP4.5, the three prominent land conversions that lead to the largest changes in the global dryland GPP are from humid to subhumid (+2.53 Pg C year^−1^; 151.1%), subhumid to humid (−0.53 Pg C year^−1^; −31.5%), and humid to semiarid (0.47 Pg C year^−1^; 28.0%) (Fig. 7). Meanwhile, droughts also cause transitions among dryland subtypes, e.g., from subhumid to semiarid, semiarid to arid lands, and so on, leading to a reduction in the GPP (Fig. 7). Degradations within semiarid (−0.37 Pg C year^−1^; −22.3%) and subhumid (−0.18 Pg C year^−1^; −10.9%), from subhumid to semiarid (−0.17 Pg C year^−1^; −10.0%), and from semiarid to arid lands (−0.07 Pg C year^−1^; −4.2%) cause the substantial reductions in GPP (Fig. 7). Our results indicate that the increase in dryland GPP due to expansion of subhumid and semiarid dryland subtypes into previously humid regions significantly exceeds the decreased dryland GPP from drought-induced dryland degradation and the (less common) contraction of the subhumid subtype due to conversion to humid lands. This leads to an overall increase in regional and global dryland GPP in this century.

## Discussion

Global drylands will experience substantial expansion, degradation, and conversions among dryland subtypes under future climate change, leading to changes in dryland ecosystem structures and functioning and thus GPP. Indeed, changes in dryland ecosystems have already happened over the past decades. For example, vegetated drylands have exhibited large-scale conversions into bare ground in the last 30 years^27^. These conversions have various causes; e.g., in the southwestern United States, they are probably the consequence of the increased dominance of invasive species^28^. Long-term precipitation reductions in growing seasons have caused the reduced vegetated drylands in Australia^29^. In the Mongolian steppe, however, the extensive GRA deterioration was attributed to a combined effect of rising air temperatures, reduction in rainfall, and overgrazing^30^. Dryland expansion acts as the primary process regulating future global dryland GPP trends and variability. Under RCP4.5, East Asia will replace North America as the largest contributor to global dryland GPP trends, whereas Australia remains to contribute the largest to the global dryland GPP IAV in this century. Although the semiarid subtype continues to dominate global dryland GPP variability, the arid subtype and the subhumid subtype play an increased and a decreased role in regulating the global dryland GPP variability, respectively. Our results on the global dryland GPP trends and IAV under RCP4.5 are conservative, and global dryland GPP spatiotemporal variability will be likely to increase if future climate change follows the RCP8.5 scenario (Supplementary Fig. 9B). Indeed, the dryland GPP under RCP8.5 shows a 25% increase by the end of this century relative to the 2000–2014 baseline due to a larger dryland expansion rate (Fig. 5). The primary trend of global dryland GPP hotspots shift from East Asia in 2011–2070 to North America in 2071–2100, while North America and East Asia, instead of Australia, play the most important role in regulating the global dryland GPP IAV in 2041–2100 (Supplementary Fig. 10, Supplementary Tables 6 and 7). The semiarid subtype in Africa and East Asia will lead to enhanced GPP variability in the global drylands (Supplementary Fig. 11). Our analysis demonstrates that regional and subtype contributions to the global dryland GPP trend and IAV are subjected to substantial shifts under future climate change.

Note that because this study is based on historical MODIS GPP datasets and the derived GPP, it does not account for potential dynamic responses of dryland ecosystems to future climate change. Global dryland ecosystems would likely experience more drastic changes in their structures and functioning (e.g., changes in water use efficiencies) under multiple elevated climatic and environmental stresses such as droughts, heat waves, grazing, invasions of exotic species, CO_2_ fertilization, woody encroachment, adaptations, and disturbances^31–34^. All of these processes induce lagged and nonlinear effects on dryland ecosystem–climate interactions. Evidence shows that lag effects can be attributed to complex interacting feedbacks such as lagged respiration of soil biota, carryover effects of antecedent climate responses (e.g., water deficit), and changes in nutrient cycles^35–40^. Variability in lag effects may be particularly significant in water-limited dryland ecosystems where precipitation events have strong impacts on the variability in aboveground NPP, soil N availability, and drought sensitivity^39^. It was found that lag effects can explain between 18 and 28% of the response variables in semiarid and arid ecosystems^41^. However, limited or no lag effects are simulated by current climate-vegetation models^38^. This inadequate quantitative understanding of lagged responses to climate extreme is of particular concern because a new climate regime with more frequent and intense extreme events could perpetuate the lag effects, leading to large bias in predicting climate-carbon feedbacks^35^. However, the lag effects are not quantified and discussed in our study. Thus, uncertainties and limitations are expected in projected spatiotemporal variabilities in global dryland GPP. Nevertheless, our MODIS-derived GPP over global drylands show variabilities comparable with previous studies and those obtained by models (e.g., CMIP5) and empirical observation-based datasets. In addition, since precipitation and air temperature explained the majority of dryland GPP variability (79%), other environmental drivers such as changes in ecosystem types play relatively small roles in regulating dryland GPP variability. Thus, our projected GPP variability in 2010–2100 should capture its major spatiotemporal features under future climate change. Dynamic Earth system models that couple the physical, biophysical, societal, economic, and biogeochemical processes that govern long-term global dryland ecosystem carbon cycle–climate feedbacks are essential to more fully understand dryland ecosystem–climate interactions.

## Methods

### Aridity index

AI, a measure of climatic dryness, is the ratio of the annual precipitation (*P*) to annual PET. Drylands are defined as regions where the AI is less than 0.65, and humid regions are defined as the regions where the AI is greater than 0.65. We used previously published global AI datasets with a spatial resolution of 0.5° × 0.5° from 2000 to 2100 (ref. ^19^). Previous comparisons demonstrated strong agreement between CMIP5 historical simulations for 1948–2005 and observations of dryland variability^19^.

### MODIS GPP data

GPP is the capacity of vegetation to capture carbon and energy during the process of photosynthesis^42^. We used monthly MODIS global terrestrial GPP products from 2000 to 2014 with a spatial resolution of 0.5° × 0.5° (ref. ^23^). The MODIS17 is the first continuous satellite-driven dataset monitoring global vegetation productivity. The fundamentals for the MOD17 algorithm are to apply radiation conversion efficiency in predicting daily GPP. Specifically, the MODIS GPP is estimated using a light use efficiency (LUE) model developed by Monteith^42^, in which gross photosynthesis is proportional to the amount of the absorbed photosynthetically active radiation (PAR) by plants. The LUE model is rewritten as^43,44^

1 $${\mathrm{GPP}} = \varepsilon _{\mathrm{max}} \times 0.45 \times {\mathrm{SW}}_{{\mathrm{rad}}} \times f{\mathrm{PAR}} \times f{\mathrm{VPD}} \times {{fT}}_{{\mathrm{min}}},$$

where *ε*_max_ is the maximum LUE, SW_rad_ is the downward solar radiation, of which 45% is PAR, *f*PAR is the fraction of PAR being absorbed by plants, and *f*VPD and *f*T_min_ are the reduction scalars from water stresses (high daily VPD) and low daily minimum temperature. Varying climate conditions and diverse plant functional types pose difficulties in accessing the uncertain of MODIS GPP data. One primary uncertainty source is due to lack of accurate definition of $\varepsilon$, especially for complex and diverse ecosystems for which the same $\varepsilon$ could induce large uncertainties in estimating GPP. GPP can be overestimated for ecosystems with low productivity and underestimated for ecosystems with high productivity^45^. In arid and semiarid regions, accounting for the effect of soil moisture in the algorithm may reduce the uncertainty in GPP over drylands^46^. In addition, such LUE model has known issues in modeling fluxes in dry sites, particularly when soil moisture and VPD are decoupled and VPD is not a good indicator of water availability and water stress. Nevertheless, the MODIS GPP product remains the most widely used global GPP product with its advantage of continuously spatial and temporal coverage^47^.

MODIS products (Levels 1, 2, 3, and 4) are a new type of integerized sinusoidal projection data. We used MODIS Re-projection Tools to convert the MODIS GPP data formats and map projections to the WGS84/geographic system^48^. The global GPP datasets from 2000 to 2014 were used in this study.

### FLUXNET GPP data

We compared the MODIS-derived GPP with the FLUXNET GPP. The FLUXNET GPPs are based on eddy flux measurements and have been widely used to validate MODIS GPP^49^. FLUXNET has established over 800 long-term eddy covariance flux tower sites, ranging from 30°S to 70°N, covering a wide range of climatic zones and terrestrial ecosystems (http://www.fluxnet.ornl.gov/). FLUXNET employs standard data quality assurance and control, and postfield data processing procedures to ensure high quality flux datasets with significantly reduced uncertainties associated with site-to-site variations in fluxes. The FLUXNET GPPs are obtained from the difference between measured net ecosystem exchange and calculated ecosystem respiration (Reco)^45^

2 $${\mathrm{GPP}} = {\mathrm{NEE}} - {\mathrm{Reco}},$$

where the partitioning for Reco uses the nighttime method^49,50^.

In this study, we compared MODIS-derived GPP with flux-derived GPP from 13 FLUXNET flux sites across five different biomes across the global (e.g., SAV, GRA, WSA, CRO, and OSH). The flux data covered the years after 2000, available from 1 to 14 years for the selected sites (Table S2) after passing FLUXNET quality control. The results show that the MODIS GPP is well correlated with FLUXNET GPP (Supplementary Fig. 12; Supplementary Table 8). Our comparisons confirm the quality of MODIS datasets for studying temporal and spatial variations of the dryland GPP.

### FLUXCOM GPP data

We also compared the spatial variations in the annual global dryland GPP from the MODIS datasets with those from the FLUXCOM datasets (Supplementary Figs. 13 and 14; Supplementary Table 9). The FLUXCOM GPP datasets (http://www.fluxcom.org/) are derived with upscaling approaches based on three machine learning algorithms that integrate 224 FLUXNET site level observations, satellite remote sensing, and meteorological data. The use of three machine learning algorithms minimizes sources of uncertainty in empirical upscaling and ultimately provides an ensemble of machine learning-based global flux products to the scientific community for evaluating process-based land-surface models [http://www.fluxcom.org/]^51^. Briefly, the machine learning algorithms were initially trained to site-level observations of the explanatory climate and land-surface variables. To capture variabilities in vegetation greenness and land-surface temperature with reliable input of changes associated with soil moisture, the variables from high-resolution satellite remote sensing data were fed into the extensive variable selection analysis^52^. The machine learning algorithms and their training and a thorough cross-validation of the data are presented in Tramontana et al.^53^. To obtain the global GPP, the trained and validated machine learning algorithms were driven by with global gridded satellite data of selected explanatory variables, at 10 km spatial and 8 days temporal resolutions for the period 2000–2014. Due to the complex topography and ecosystem functions, large uncertainties remain in obtaining spatial and temporal patterns of GPP on regional and global scales from FLUXNET GPP using different upscaling methods^54,55^. Since the 224 flux sites are not uniformly distributed over the globe with different climate types and plant function types, and there are less dense flux sites over drylands, caution should be also taken when explaining CO_2_ dynamics over drylands.

### CMIP5 GPP data

We also compared the MODIS-derived GPP with the simulated GPP from 15 CMIP5 models. The CMIP5 simulations include long-term (century timescale) integrations and near-term integrations (10–30 years) or decadal prediction experiments^25^. The CMIP5 GPP datasets can be downloaded at https://esgf-node.llnl.gov/search/esgf-llnl/. We compared our GPP with the ensemble means of the GPP from 2010 to 2100 simulated by the 15 CMIP5 models (Supplementary Table 8). The multimodel ensemble, which is different from the ensemble of simulations produced by individual models, represents a variety of best-effort attempts to simulate the climate system. To the extent that these attempts are at least somewhat independent and that the collection of models is not systematically biased on the whole, the ensemble can be used to provide both a consensus representation of the climate system and, based on the spread of model results, provide some measure of how much confidence might be placed in that consensus.

### Meteorological data

The precipitation dataset from the National Center for Environmental Prediction’s (NCEP) Climate Prediction Center is an observation-based dataset with a global latitude-longitude resolution of 0.5° × 0.5° (ref. ^56^), which can be downloaded at http://www.cpc.ncep.noaa.gov/. Air temperature, VPD, and wind speed (WS) are obtained from the NCEP/National Center for Atmospheric Research reanalysis^57^, which can be downloaded at https://www.esrl.noaa.gov/psd/data/gridded/reanalysis/. The soil moisture data used here are the monthly GLDAS version 2 product (GLDAS-2) during the period from 2000 to 2014, with a horizontal resolution of 1° × 1° (ref. ^58^), which can be accessed at https://disc.gsfc.nasa.gov/datasets?keywords=GLDAS. PET is estimated using the Penman–Monteith algorithm, which is driven by meteorological parameters including net radiation, soil heat flux, mean daily air temperature, WS, saturation vapor pressure, and vapor pressure^21^. These meteorological data from 2000 to 2014 are used to analyze their influence on GPP.

### Statistical analysis

To show the accuracy of MODIS product, we computed the correlation coefficients using the ordinary least square (OLS) regression and the root mean squared error (RMSE) between the two GPP products,

3 $${\mathrm{RMSE = }}\sqrt {\frac{{\mathop {\sum }\nolimits_{t = 1}^n \left( {{\mathrm{GPP}}_{{\mathrm{flux}}} - {\mathrm{GPP}}_{{\mathrm{MODIS}}}} \right)^2}}{n}} ,$$

where GPP_flux_ and GPP_MODIS_ are the FLUXNET-derived and MODIS-derived GPP, respectively. The linear trend of GPP was estimated using the OLS regression, and the significance level of the linear correlation was calculated with a two-tailed test. The IAV of GPP was computed as the mean absolute global GPP anomaly.

### Method to calculate relative contributions

The relative contributions of the individual GPP trend from each of the eight regions to the global dryland GPP trend were calculated such that the overall trend equals the sum of the trend for each of the eight regions. To calculate the relative contributions of the GPP IAV from each of the eight regions to the global dryland GPP IAV, we adopted an index used in a previous study^10^

4 $$f_i = \mathop {\sum }\limits_t \frac{{x_{it}\left| {X_t} \right|}}{{X_t}}/\mathop {\sum }\limits_t \left| {X_t} \right|,$$

where *x*_*it*_ is the GPP anomaly for region *i* at time *t* in years, and *X*_*t*_ is the global dryland GPP anomaly, so that $X_t = {\mathrm{\Sigma }}_tx_{jt}$. The *f*_*i*_ is the average relative anomaly $x_{it}/X_t$ for region *i*, and this definition should guarantee that ${\mathrm{\Sigma }}_if_i = 1$. The resulting scores for a region *f*_*i*_ represent its contribution to global dryland variations. Regions with high scores contribute strongly to overall dryland GPP variations, while regions with low scores contribute less. Regions with negative scores dampen variations.

## Supplementary information

Supplementary Information

## Data Availability

All processed GPP data used in this study are available at 10.6084/m9.figshare.10271447. Other data can be downloaded in the websites provided in the “Methods” section. Additional data that support the findings of this study are available from H.L. upon reasonable request.
