# Supplementary material for: Accelerated dryland expansion regulates future variability in dryland gross primary production
Source: Nat Commun. 2020 Apr 3;11:1665. doi: 10.1038/s41467-020-15515-2 (PMC7125214; doi:10.1038/s41467-020-15515-2)
Supplement: Supplementary file 1 — Supplementary Information [file 41467_2020_15515_MOESM1_ESM.pdf]

Supplementary information for

Accelerated dryland expansion regulates future variability in dryland gross primary production

By Yao et al.

Supplementary Figures

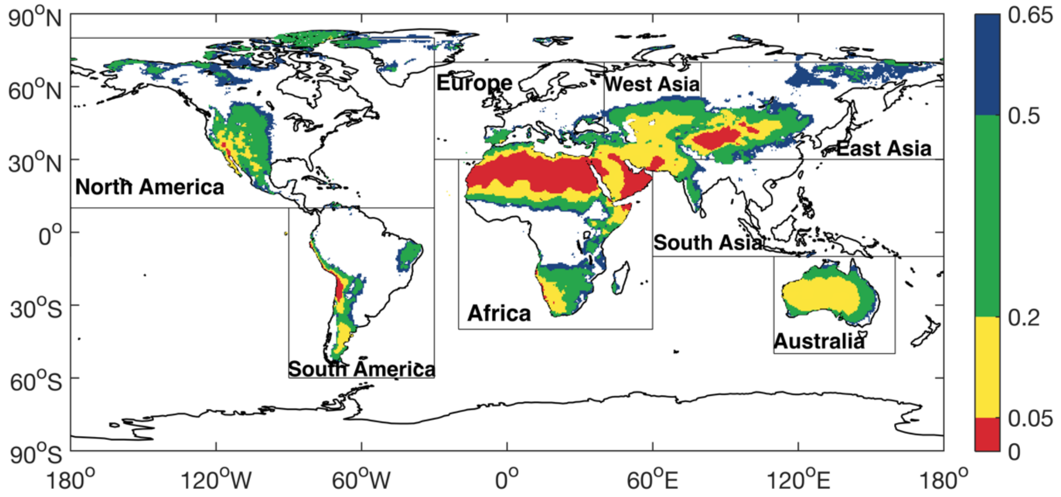

Supplementary Figure 1. Global dryland distribution according to the aridity index. Eight regions are used in this study. The global drylands are divided into four subtypes according to the aridity index (AI) ranges: hyper-arid (AI ≤ 0.05), arid (0.05 < AI ≤ 0.2), semi-arid (0.2 < AI ≤ 0.5), and dry sub-humid (0.5 < AI ≤ 0.65).

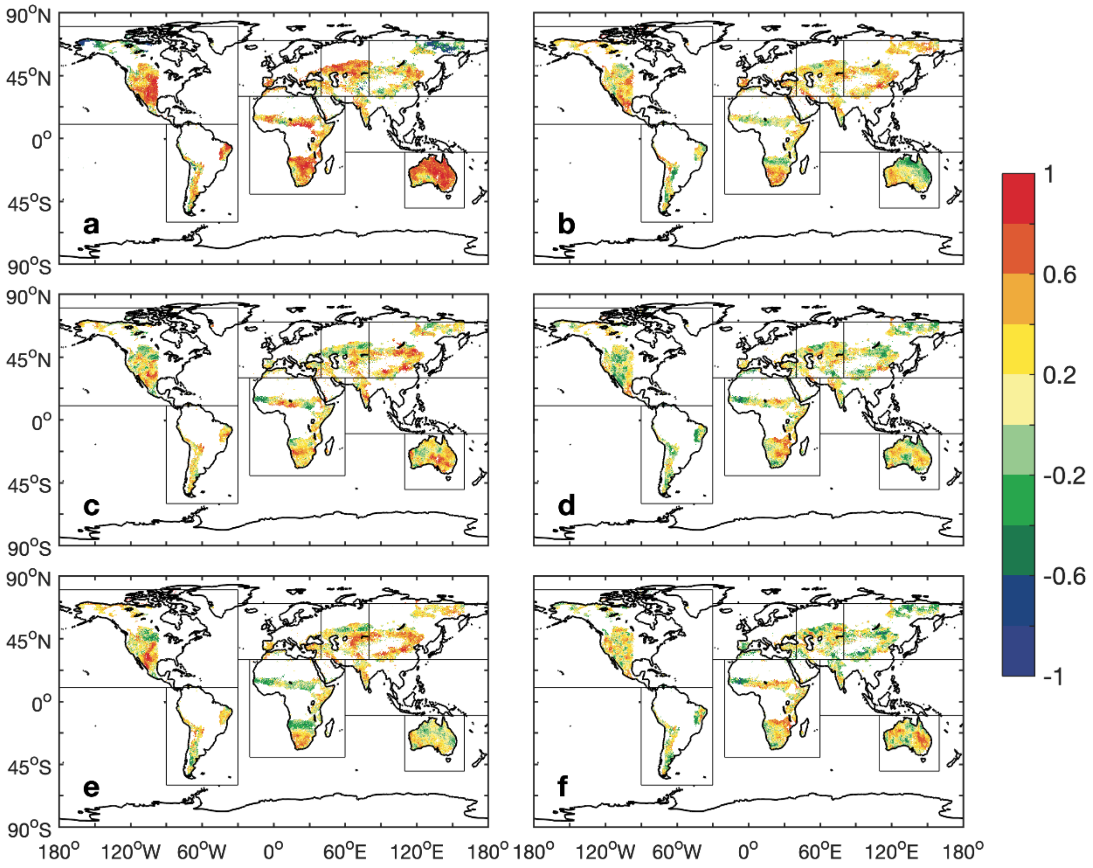

Supplementary Figure 2. Correlation coefficients between the trend of gross primary production (GPP) and the trends of environmental variables (2000–2014). **a**, Precipitation. **b**, Air temperature. **c**, Soil moisture. **d**, Sensible heat. **e**, Vapor pressure deficit. **f**, Potential evapotranspiration.

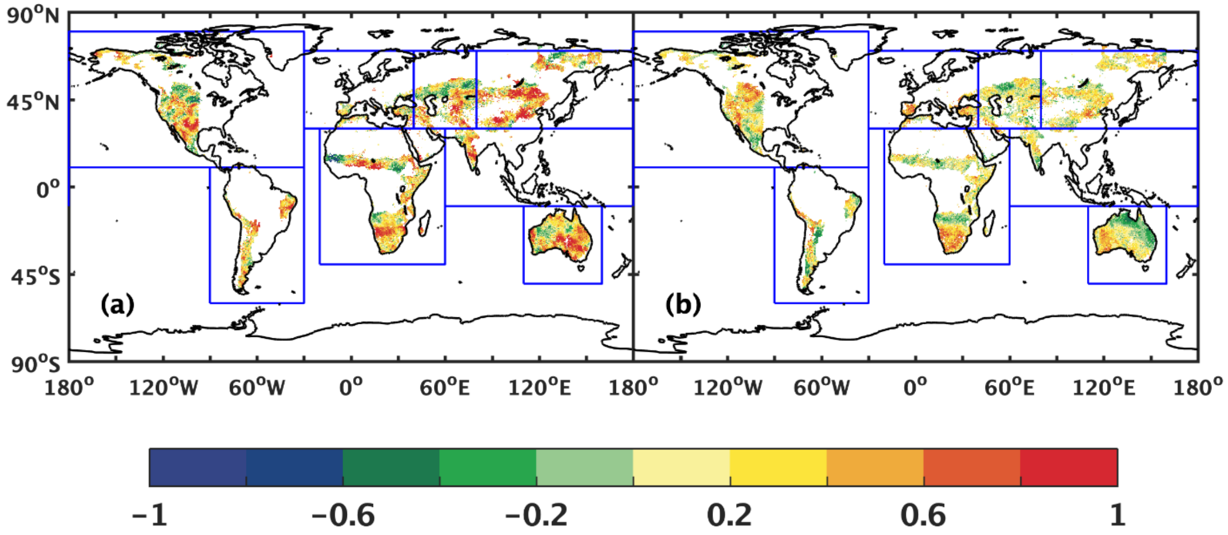

Supplementary Figure 3. The partial correlation coefficients (a) between gross primary production (GPP) and precipitation (P) and (b) between GPP and potential evapotranspiration (PET) for the period from 2001 to 2014. We used partial correlation to calculate the correlation coefficients between the dryland GPP and environmental variables (P and PET). Our results show that precipitation and temperature are the two most important variables in influencing GPP variability. We then used partial correlation to analyze the effects of P (see panel **a**) and PET (see panel **b**) on the dryland GPP. In drylands, the GPP is primarily positively correlated with P, which indicates that an increase in P leads to the plant growth. There are spatial differences in the correlation between GPP and PET. In the south of North America, Central Asia, south of Africa and the north of Australia, negative correlation between GPP and PET is primarily present. While in some high-latitude drylands in Northern Hemisphere, GPP is positively related to PET. Our results indicate that the dryland GPP is mainly affected by P, and the area affected by PET is relatively small.

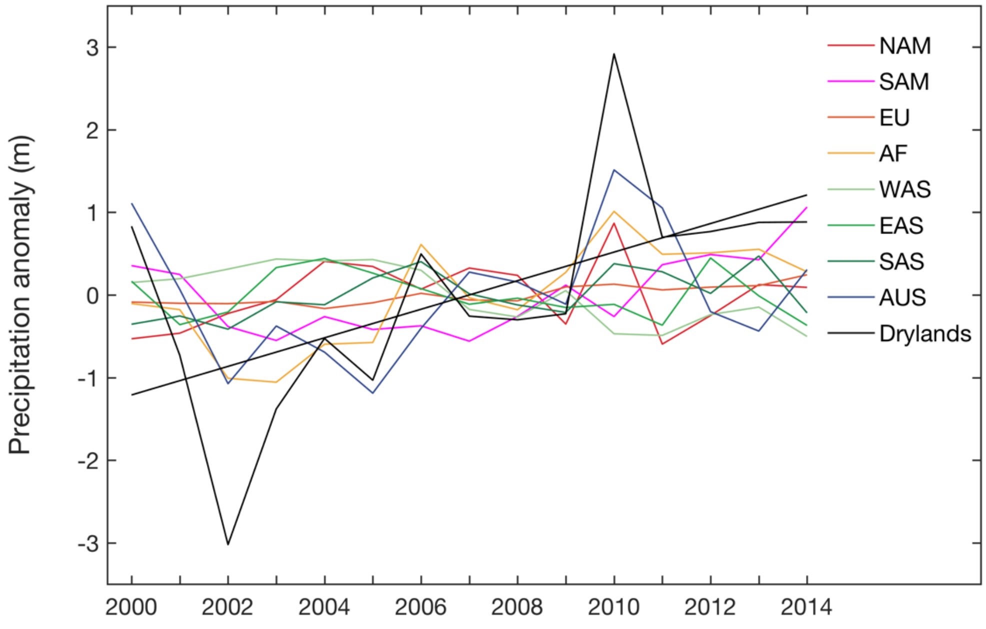

Supplementary Figure 4. Annual precipitation anomaly in the eight dryland regions (2000–2014). The black line denotes the linear trend of precipitation in global drylands. NAM: North America, SAM: South America, EU: Europe, AF: Africa, WAS: West Asia, EAS: East Asia, SAS: South Asia, AUS: Australia, Drylands: the global drylands.

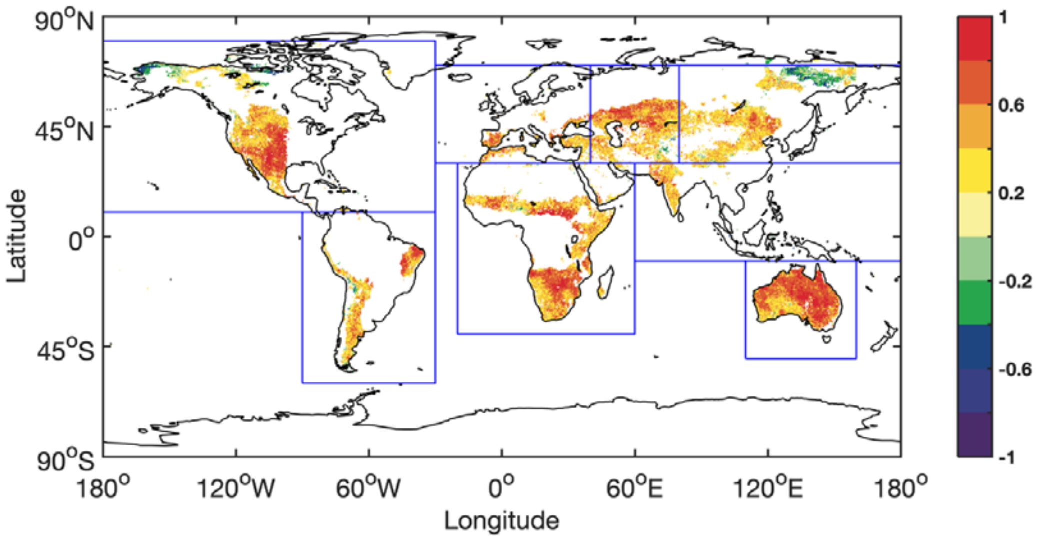

Supplementary Figure 5. Correlation coefficients between the gross primary production (GPP) and the aridity index (AI) (2000–2014).

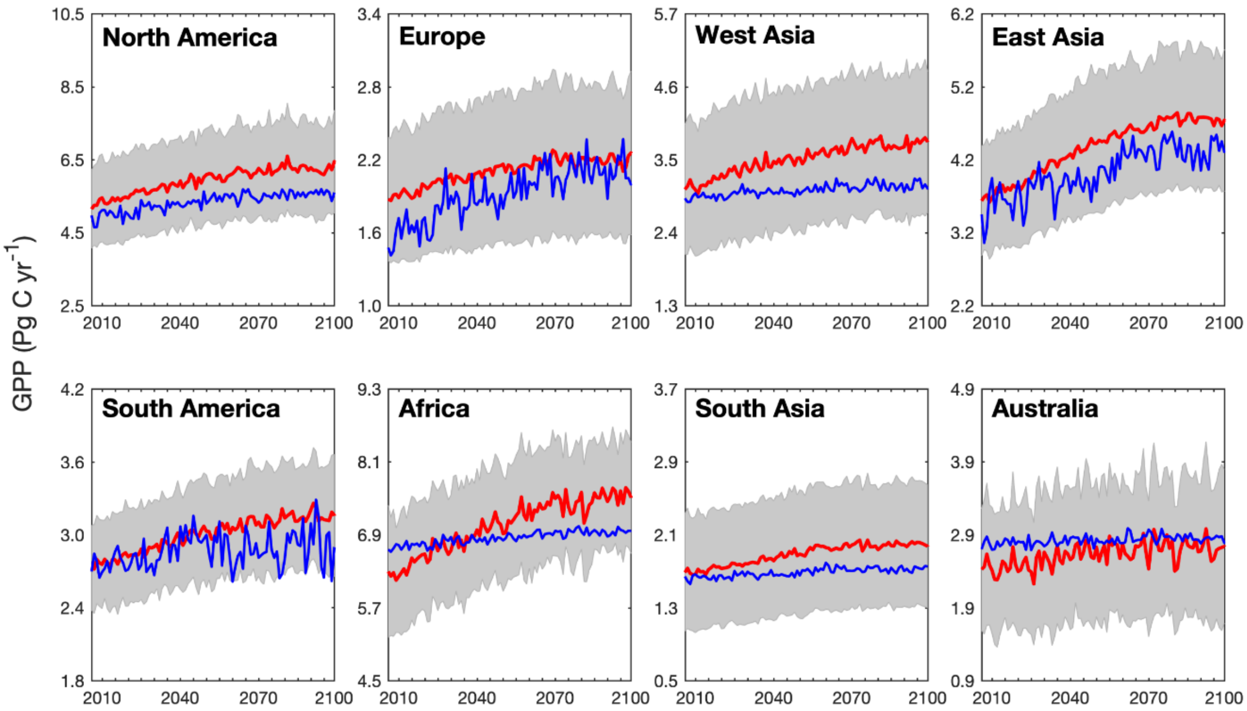

Supplementary Figure 6. Variations in GPP in the eight regions from 2010 to 2100. Red lines denote the ensemble means of the 15 global climate modeling results of CMIP5. Gray shaded areas denote one standard deviation of the modeling results. Blue lines denote the derived gross primary production (GPP) from the equations in Supplementary Table 6.

A

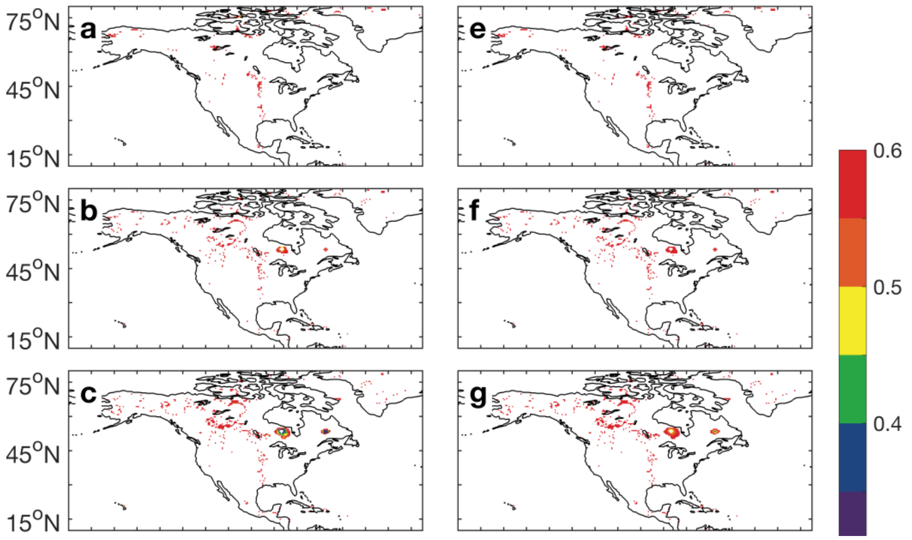

B

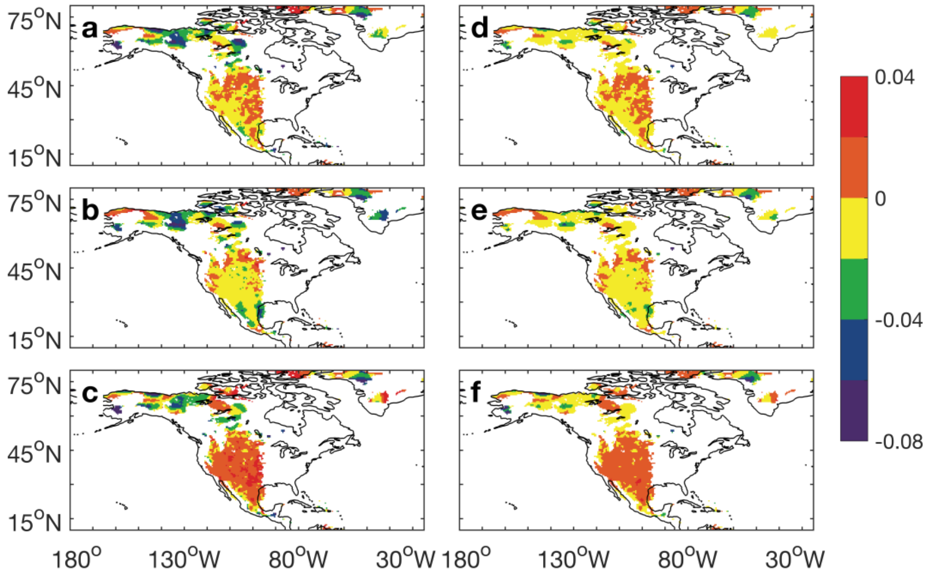

Supplementary Figure 7. (A) The aridity index in the expanded drylands in North America under RCP4.5: a, 2011–2025. **b**, 2026–2040. **c**, 2041–2055. **d**, 2056–2070. **The gross primary production (GPP) in the expanded drylands in North America: e**, 2011–2025. **f**, 2026–2040. **g**, 2041–2055. **h**, 2056–2070. **(B) Changes in aridity index in the historical drylands in North America under RCP4.5: a**, 2026–2040 relative to 2011–2025. **b**, 2041–2055 relative to 2026–2040. **c**, 2056–2070 relative to 2041–2055. **Changes in the historical drylands in North America: d**, 2026–2040 relative to 2011–2025. **e**, 2041–2055 relative to 2026–2040. **f**, 2056–2070 relative to 2041–2055.

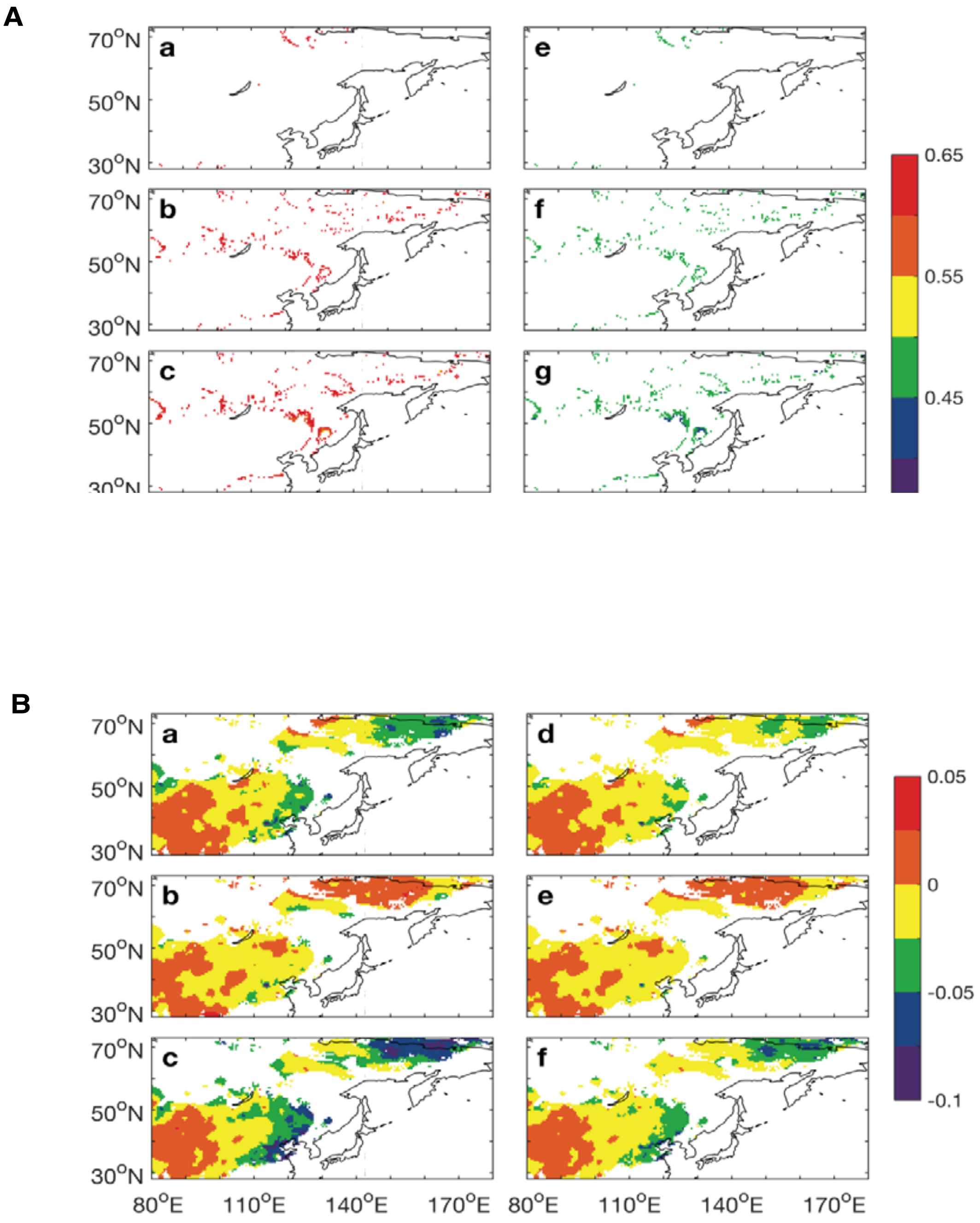

Supplementary Figure 8. (A) The aridity index in the expanded drylands in East Asia under RCP4.5: **a**, 2011–2025. **b**, 2026–2040. **c**, 2041–2055. **d**, 2056–2070. **The gross primary production (GPP) in the expanded drylands in East Asia: e**, 2011–2025. **f**, 2026–2040. **g**, 2041–2055. **h**, 2056–2070. **(B) Changes in aridity index in the historical drylands in East Asia under RCP4.5: a**, 2026–2040 relative to 2011–2025. **b**, 2041–2055 relative to 2026–2040. **c**, 2056–2070 relative to 2041–2055. **Changes in the historical drylands in East Asia: d**, 2026–2040 relative to 2011–2025. **e**, 2041–2055 relative to 2026–2040. **f**, 2056–2070 relative to 2041–2055.

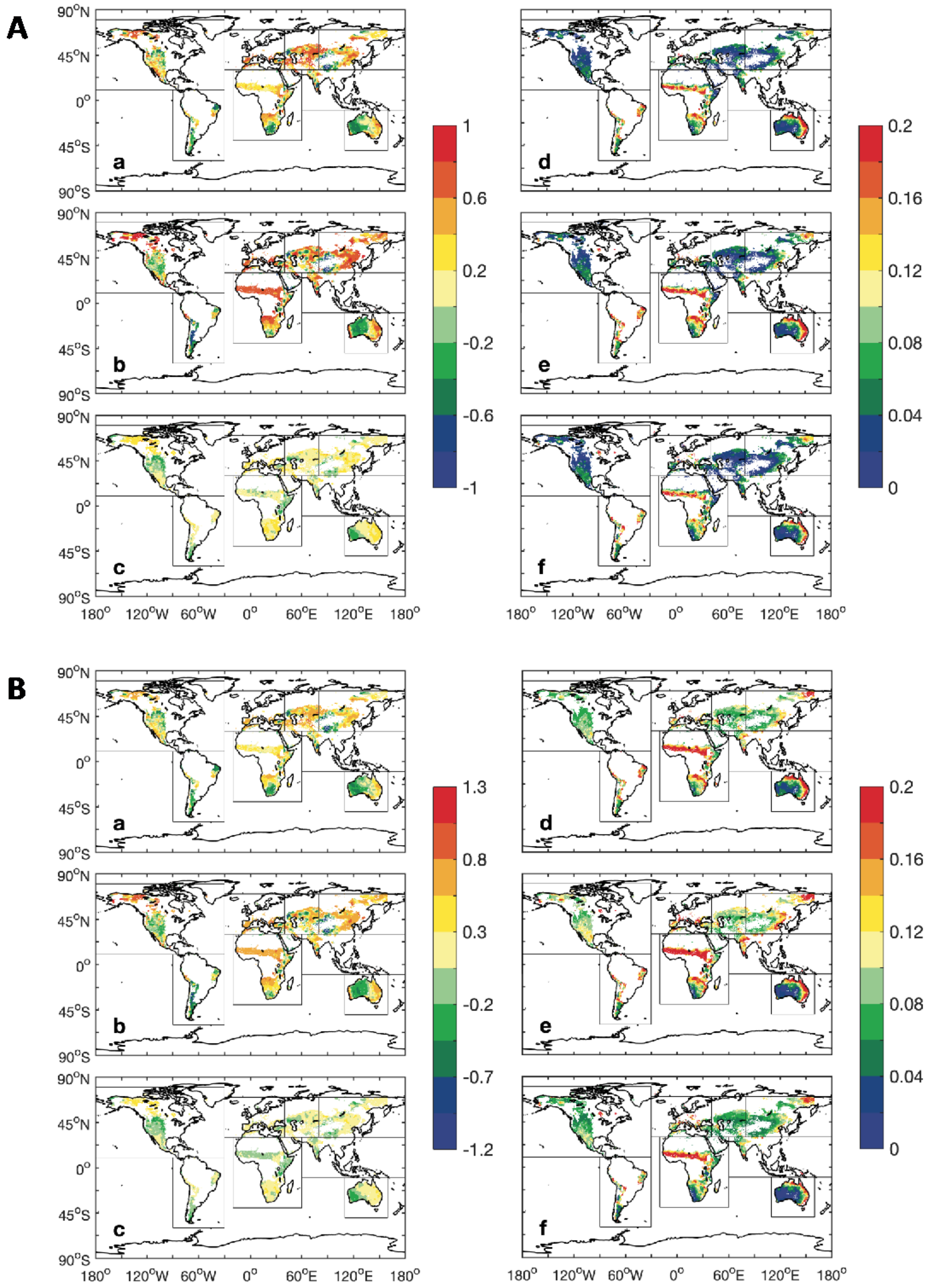

Supplementary Figure 9. Global dryland gross primary production (GPP) (a-c) trends and (d-f) interannual variability (IAV) under (A) RCP 4.5 and (B) RCP8.5 during the three periods: (a and d) 2011-2040, (b and e) 2041-2070, and (c and f) 2071-2100.

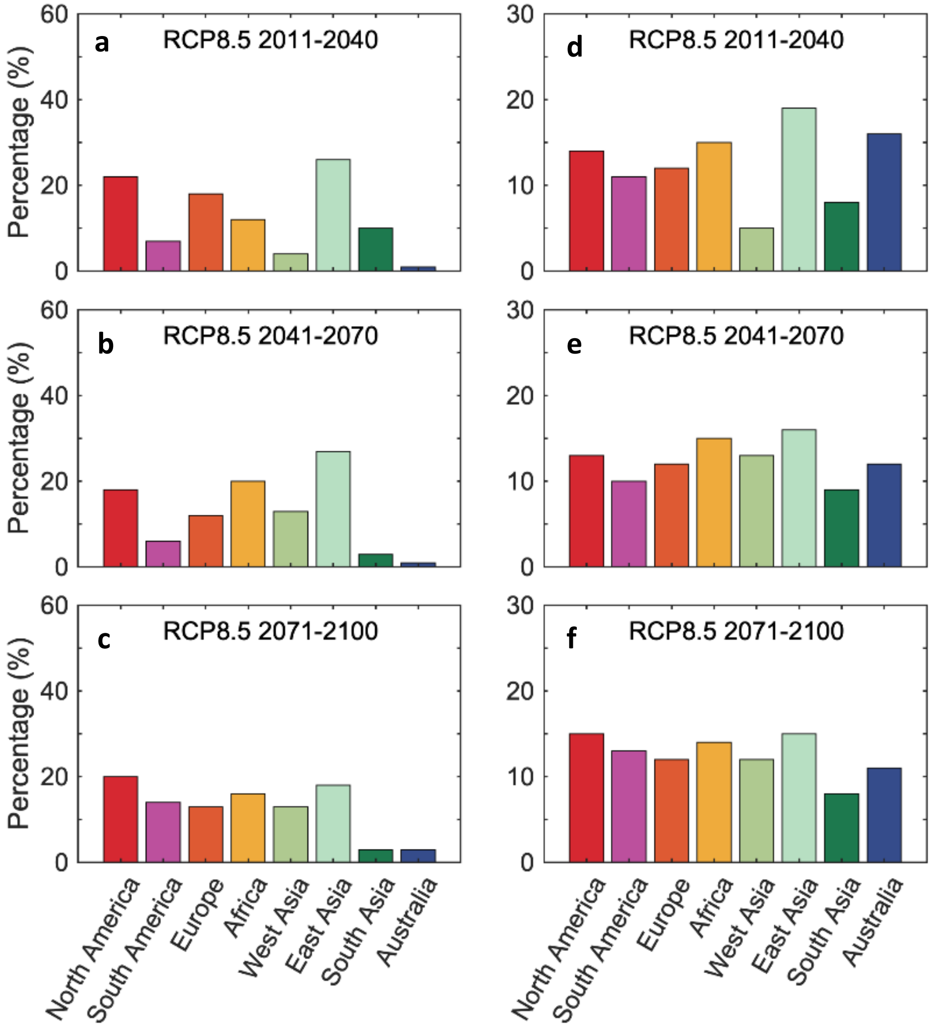

Supplementary Figure 10. Relative contributions from the eight regions to the global dryland gross primary production (GPP) (a-c) trend and **(d-f)** interannual variability (IAV) during the three periods in 2011–2100 (RCP8.5). NAM: North America, SAM: South America, EU: Europe, AF: Africa, WAS: West Asia, EAS: East Asia, SAS: South Asia, AUS: Australia, Drylands: the global drylands.

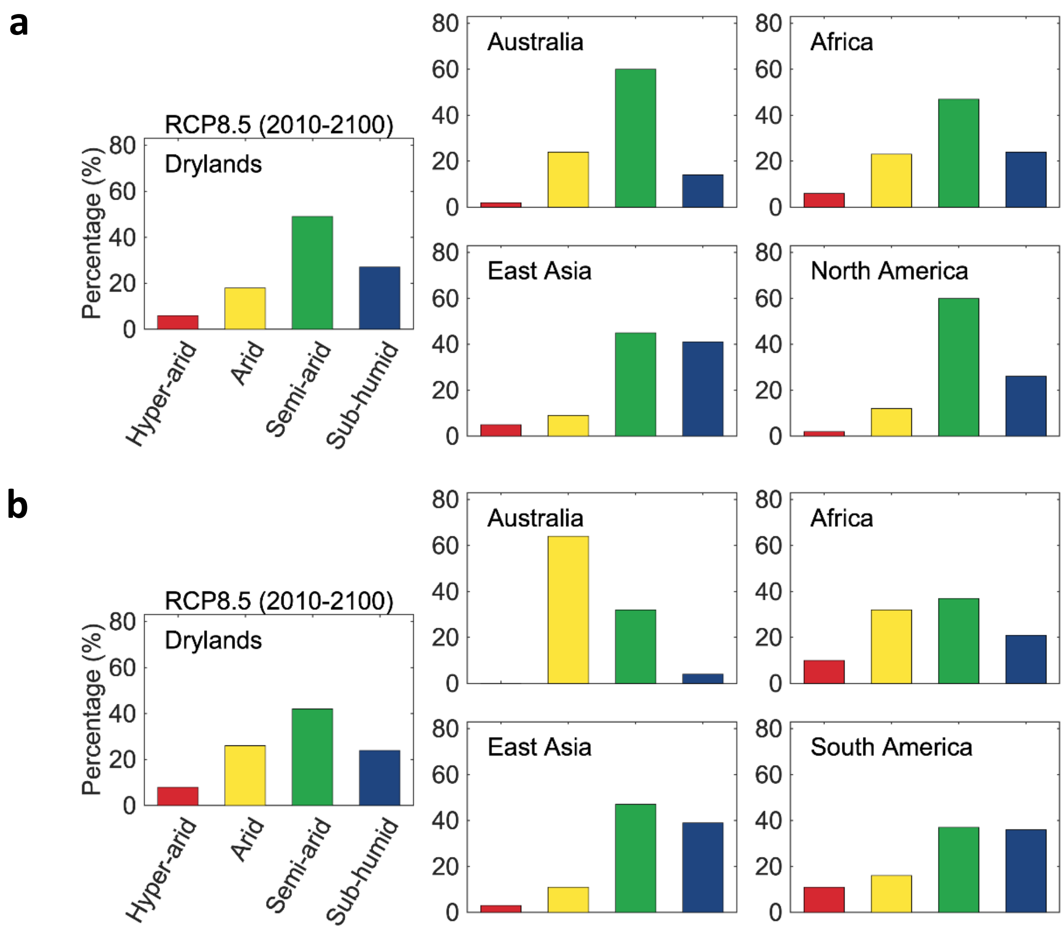

Supplementary Figure 11. Relative dryland subtype contributions to the global dryland gross primary production (GPP) under RCP8.5. (a) trend and **(b)** interannual variability (IAV).

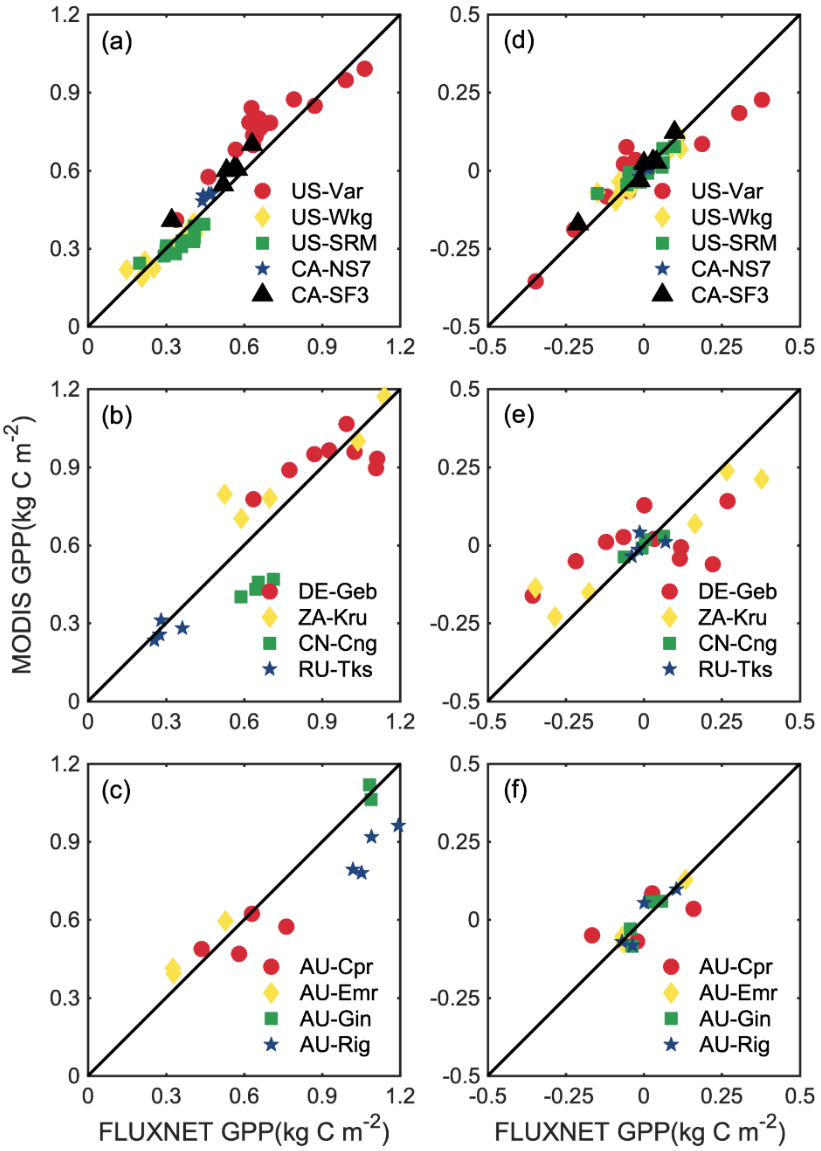

Supplementary Figure 12. Comparison between the MODIS-derived GPP and the FLUXNET GPP in 13 dryland sites. The FLUXNET 13 flux observation sites are located across the globe in five different biomes: savanna (SAV), grassland (GRA) and woody savannas (WSA), croplands (CRO) and open shrublands (OSH). The flux data are available from one to fourteen years for the selected sites since 2000 (Supplementary Table 2) after passing the FLUXNET quality control criteria. **a–c,** Correlation of annual MODIS gross primary production (GPP) and annual FLUXNET GPP. They are well correlated, consistent with the results of Zhao et al[1]. **d–f,** A comparison of the annual GPP anomaly with respect to its multiple-year means between the MODIS GPP and the FLUXNET GPP. The comparisons indicate that the MODIS GPP data can capture the GPP interannual variability (IAV).

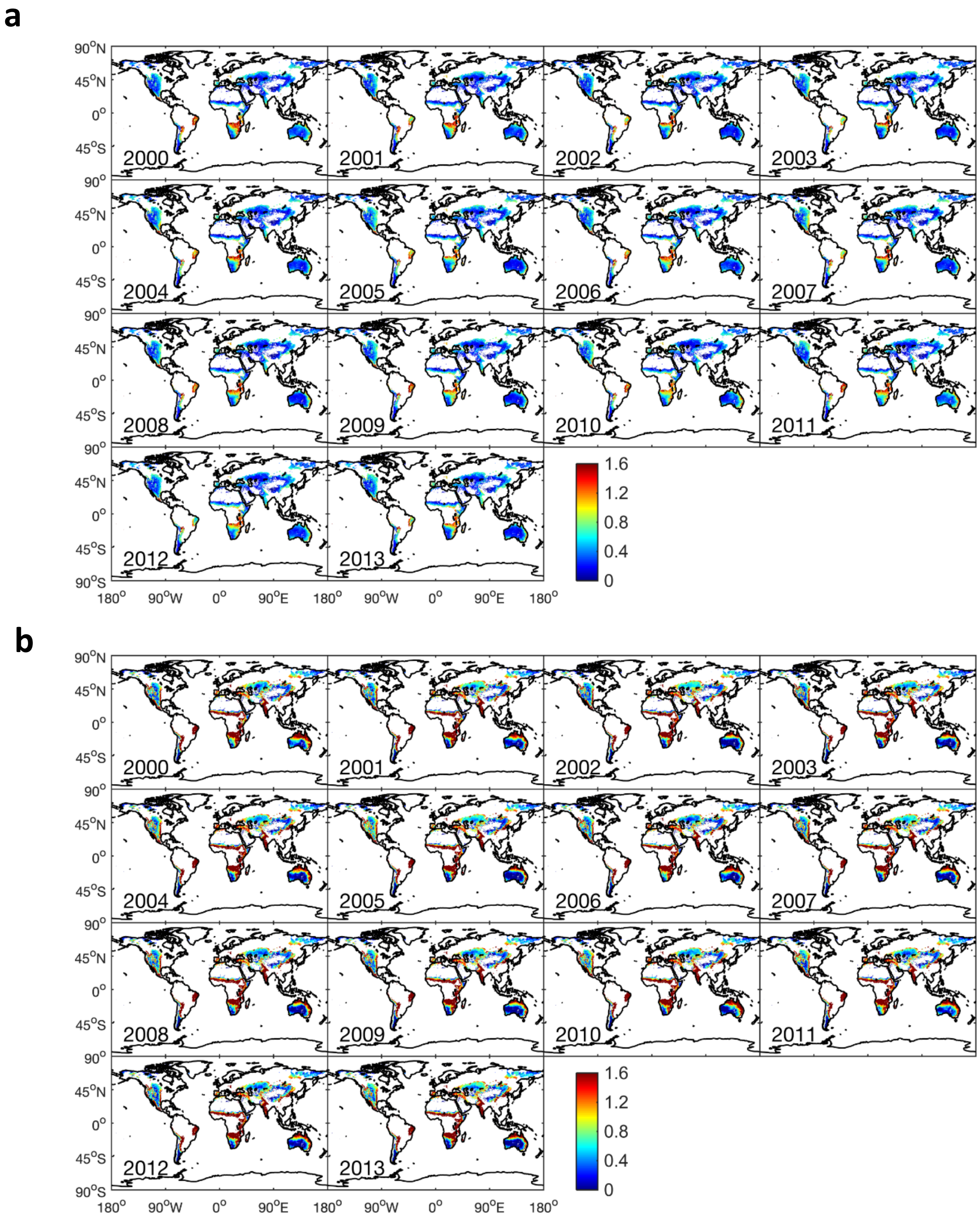

Supplementary Figure 13. The spatial distributions of the annual mean dryland GPP from 2000 to 2013. (a) MODIS gross primary production (GPP). **(b)** FLUXCOM GPP (http://www.fluxcom.org/). The global FLUXCOM GPP is derived with upscaling approaches based on machine learning methods that integrate FLUXNET site-level observations, satellite remote sensing, and meteorological data.

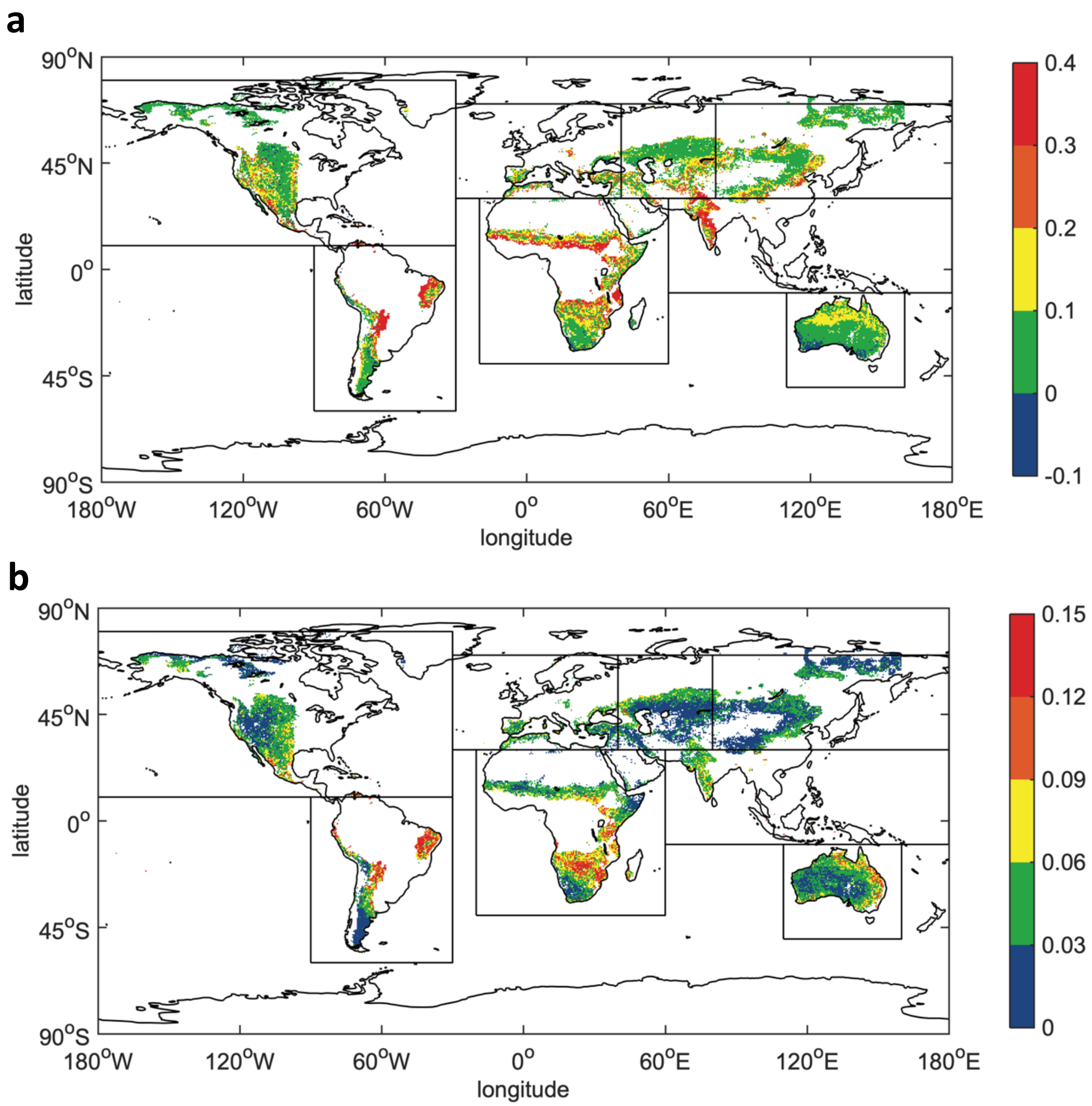

Supplementary Figure 14. (a) The mean and (b) standard deviation for the difference between the FLUXCOM GPP and MODIS GPP. More than 85% of global drylands have greater FLUXCOM gross primary production (GPP) than MODIS GPP. About 80% of drylands show a difference of less than 10%. In some areas in South America, Africa, and Asia, MODIS GPP is 30% lower than FLUXCOM GPP. Except in some areas in South America, Africa, and eastern Australia, the difference between the two datasets has small spatial variations. The mean and standard deviation for the difference between the MODIS GPP and FLUXCOM GPP in the eight regions are provided in Supplementary Table 3.

Supplementary Tables

Supplementary Table 1. Latitude/longitude information for the eight regions.

	Region	Latitude	Longitude
1	North America (NAM)	10^oN-80^oN	180^oW-30^oW
2	South America (SAM)	60^oS-10^oN	180^oW-30^oW
3	Europe (EU)	30^oN-70^oN	30^oW-40^oE
4	Africa (AF)	60^oS-30^oN	30^oW-60^oE
5	West Asia (WAS)	30^oN-70^oN	40^oE-80^oE
6	East Asia (EAS)	30^oN-70^oN	80^oE-180^oE
7	South Asia (SAS)	10^oS-30^oN	40^oE-180^oE
8	Australia (AUS)	50^oS-10^oS	110^oE-160^oE

Supplementary Table 2. Relative contribution of the regional dryland gross primary production (GPP) trend to the global dryland GPP trend (RCP4.5)

	North America	South America	Europe	Africa	West Asia	East Asia	South Asia	Australia
2000-2014 (MODIS)	45%	-10%	9%	18%	-4%	21%	3%	18%
2011-2040	9%	1%	10%	16%	17%	27%	18%	2%
2041-2070	7%	-2%	8%	18%	13%	35%	16%	5%
2071-2100	16%	8%	13%	5%	16%	21%	11%	10%
2011-2100	15%	5%	10%	10%	9%	32%	12%	7%

Supplementary Table 3. Relative contribution of the regional dryland gross primary production (GPP) interannual variability (IAV) to the global dryland GPP IAV (RCP4.5)

	North America	South America	Europe	Africa	West Asia	East Asia	South Asia	Australia
2000-2014 (MODIS)	8%	20%	10%	15%	6%	6%	10%	25%
2011-2040	7%	17%	11%	17%	5%	11%	9%	23%
2041-2070	8%	17%	12%	16%	4%	11%	10%	22%
2071-2100	9%	15%	14%	16%	4%	10%	11%	21%
2011-2100	9%	13%	12%	15%	7%	12%	11%	21%

Supplementary Table 4. The fitting equation between the dryland gross primary production (**GPP) and aridity index (AI) in each of the eight regions.** One asterisk indicates that the equation exceeds the 95% confidence level, and two asterisks indicate that the equation exceeds the 99% confidence level.

**Region**	**Equation**	**R^2**
North America	$GPP_{NA} = 1.70 \times AI^3 - 2.81 \times AI^2 + 1.86 \times AI + 0.12$	0.88*
South America	$GPP_{SAM} = -2.22 \times AI^3 + 2.61 \times AI^2 + 1.09 \times AI + 0.14$	0.94**
Europe	$GPP_{EU} = -4.74 \times AI^3 + 6.88 \times AI^2 - 1.81 \times AI + 0.55$	0.82*
Africa	$GPP_{AF} = -1.85 \times AI^3 + 2.31 \times AI^2 + 1.01 \times AI + 0.14$	0.98**
West Asia	$GPP_{WA} = -1.56 \times AI^3 + 1.93 \times AI^2 + 0.19 \times AI + 0.13$	0.97**
East Asia	$GPP_{EA} = -0.81 \times AI^3 + 1.14 \times AI^2 + 0.17 \times AI + 0.12$	0.96**
South Asia	$GPP_{SAS} = -0.91 \times AI^3 + 1.68 \times AI^2 + 0.39 \times AI + 0.20$	0.93**
Australia	$GPP_{AU} = -3.13 \times AI^3 + 5.49 \times AI^2 - 0.35 \times AI + 0.24$	0.92**

Supplementary Table 5. CMIP5 models used in this study

Model name	Modeler	Resolution (lon × lat)
Bcc-csm1-1	Beijing Climate Center, China	128×64
BNU-ESM	College of Global Change and Earth System Science, Beijing Normal University	128×64
CanESM1	Canadian Centre for Climate, Canada	128×64
CCSM4	National Center for Atmospheric Research, USA	288×192
GFDL-ESM2G	Geophysical Fluid Dynamics Laboratory	144×90
GFDL-ESM2M	Geophysical Fluid Dynamics Laboratory	144×90
GISS-E2-R	NASA Goddard Institute for Space Studies, USA	144×90
HadGEM2-CC	Met Office Hadley Centre, UK	192×145
HadGEM-ES	Met Office Hadley Centre, UK	192×145
IPSL-CM5A-LR	Institute Pierre-Simon Laplace, France	96×96
IPSL-CM5A-MR	Institute Pierre-Simon Laplace, France	144×143
MIROC-ESM	Japan Agency for Marine-Earth Science and Technology, Japan	128×64
MIROC-ESM-CHEM	Japan Agency for Marine-Earth Science and Technology, Japan	128×64
MPI-ESM-LR	Max Planck Institute for Meteorology, Germany	192×96
NorESM1-M	Norwegian Climate Centre, Norway	144×96

Supplementary Table 6. Relative contribution of the regional dryland gross primary production (GPP) trend to the global dryland GPP trend (RCP8.5).

	North America	South America	Europe	Africa	West Asia	East Asia	South Asia	Australia
2011-2040	22%	7%	18%	12%	4%	26%	10%	1%
2041-2070	18%	6%	12%	20%	13%	27%	3%	1%
2071-2100	20%	14%	13%	16%	13%	18%	3%	3%
2011-2100	19%	9%	12%	20%	12%	23%	4%	1%

Supplementary Table 7. Relative contribution of the regional dryland gross primary production (GPP) interannual variability to the global dryland GPP interannual variability (RCP8.5).

	North America	South America	Europe	Africa	West Asia	East Asia	South Asia	Australia
2011-2040	14%	11%	12%	15%	5%	19%	8%	16%
2041-2070	13%	10%	12%	15%	13%	16%	9%	12%
2071-2100	15%	13%	12%	14%	12%	15%	8%	11%
2011-2100	15%	9%	10%	18%	9%	18%	7%	14%

Supplementary Table 8. FLUXNET site descriptions including locations, ecosystem types, and measurement periods

Site-ID	Site-Name	LOCATION-LAT	LOCATION-LONG	IGBP	MAP
CA-NS7	UCI-1998 burn site	56.6358	-99.9483	OSH	483.27
CA-SF3	Saskatchewan -Western Boreal, forest burned in 1989	54.09156	-106.0052	OSH	470
US-Var	Vaira Ranch-lone	38.4133	-120.9507	GRA	559
US-Wkg	Walnut Gulch Kendall Grasslands	31.7365	-109.9419	GRA	407
US-SRM	Santa Rita Mesquite	31.8214	-110.8661	WSA	380
DE-Geb	Gebesee	51.1001	10.9143	CRO	470
ZA-Kru	Skukuza	-25.0197	31.4969	SAV	547
CN-Cng	Changling	44.5934	123.5092	GRA	
RU-Tks	Tiksi	71.5942	128.8878	GRA	323
AU-Cpr	Calperum	-34.0021	140.5891	SAV	
AU-Emr	Emerald	-23.8587	148.4746	GRA	
AU-Gig	Gingin	-31.3764	115.7138	WSA	
AU-Rig	Riggs Creek	-36.6499	145.5759	GRA	

Supplementary Table 9. Mean and standard deviation (kg C m^{-2}) for the difference between the MODIS-derived gross primary production (GPP) and the FLUXCOM GPP in the eight regions.

Region	North America	South America	Europe	Africa	West Asia	East Asia	South Asia	Australia
mean±std	0.10±0.08	0.15±0.08	0.08±0.04	0.15±0.04	0.09±0.03	0.08±0.03	0.14±0.05	0.07±0.05

Supplementary Reference

1. Zhao, M., Heinsch, F. A., Nemani, R. R. & Running, S. W. Improvements of the MODIS terrestrial gross and net primary production global data set. *Remote Sens. Environ.* **95,** 164–176 (2005).
